# Risk Factors of Pulmonary Tuberculosis in Aseer Region, Saudi Arabia: A Case–Control Study

**DOI:** 10.3390/healthcare13212755

**Published:** 2025-10-30

**Authors:** Faris Saeed Alsulayyim, Abdullah Abdulmohsen Alsabaani, Mohammad Abdullah Garnan, Amna Babiker Alshash, Asim Abdelwahid Elnoor Ali, Mohammed Awthah Aldail, Mazen Ali Asiri, Faten Ali Nasser, Syed Esam Mahmood

**Affiliations:** 1 Saudi Board of Preventive Medicine, Aseer Health Cluster, Abha 62523, Saudi Arabia; 2 Department of Family & Community Medicine, College of Medicine, King Khalid University, Abha 61421, Saudi Arabiasmahmood@kku.edu.sa (S.E.M.); 3 Public Health Department, Aseer Health Cluster, Abha 62523, Saudi Arabia; 4 Tuberculosis & Leprosy Control Center, Aseer Health Cluster, Abha 62523, Saudi Arabia; shashshash86@gmail.com; 5 Khamis Mushait Health Sector, Aseer Health Cluster, Abha 62523, Saudi Arabia; 6 Tuberculosis Unit, Department of Communicable Diseases Control, Aseer Health Cluster, Abha 62523, Saudi Arabia

**Keywords:** pulmonary tuberculosis, epidemiology, risk factors, case–control research design, Saudi Arabia

## Abstract

**Background:** Tuberculosis (TB) constitutes one of the leading causes of morbidity and mortality worldwide. Due to adopted prevention measures, good public health practices, and better treatment, its incidence, prevalence, and case fatality rates steadily fell. **Objectives and Methods:** Following a case–control research design, this study aimed to explore the risk factors of pulmonary TB among patients registered in the Aseer Region, Saudi Arabia. This study included 105 active TB cases (study group) and 143 (control group) who were randomly recruited from those attending the vaccination units at Primary Healthcare Centers in Aseer. **Results:** Participants differed significantly according to their nationality (with 65.7% being Saudi in the TB group compared with 89.5% Saudi nationals in the control, *p* < 0.001); educational status (with 27.6% being university graduates in the TB group compared with 53.8% in the control, *p* < 0.001); marital status, with most TB patients being single, compared with control (70.5%, and 44.1%, *p* < 0.001); monthly income, with lower income <5000 Saudi Riyals (SRs), among TB patients than control subjects (80% and 44.1%, *p* < 0.001); and body mass index (20% of the TB patients were underweight, compared with 6.3% in the control, *p* < 0.001). Also, participants differed significantly according to their residence, with more rural residence among TB patients than control (18.1% and 7%, *p* = 0.007), and type of houses, with 84.8% of TB patients living in apartments, compared to 68.5% of the control (*p* < 0.001). The binary logistic regression model of the possible risk factors related to pulmonary TB revealed that nationality, residence, and body mass index were the only significant independent risk factors (*p* < 0.001, *p* = 0.007, and *p* < 0.001). **Conclusions:** Personal characteristics of pulmonary TB patients include being non-Saudi, less educated, not married, unemployed, with a low monthly income, and being underweight. Risk factors related to place included residing in rural areas and living in the basement of a rented apartment.

## 1. Introduction

Every year on March 24th, the global community observes “World Tuberculosis Day,” marking the anniversary of Dr. Robert Koch’s groundbreaking announcement in 1882 regarding the identification of Mycobacterium tuberculosis, the pathogen responsible for tuberculosis (TB). This milestone laid the foundation for advancements in diagnosing and managing one of the world’s most lethal infectious diseases. The primary objective of this observance is to increase awareness about TB’s profound health, social, and economic impacts, while also encouraging intensified efforts toward eradication [1].

TB remains among the top causes of illness and death worldwide [2]. Throughout the 20th century, many developed nations experienced a steady decline in TB incidence, prevalence, and mortality rates, largely due to effective prevention strategies, robust public health systems, and improved treatment options [3]. Nonetheless, this progress has been interrupted, and recent data indicate that a person succumbs to TB every 20 s globally [4].

According to Ashaba et al. [5], in 2018, approximately 10 million individuals contracted TB globally, resulting in over 1.5 million deaths. Conversely, between 2000 and 2018, timely diagnosis and treatment contributed to saving nearly 58 million lives. Despite these achievements, eliminating the TB epidemic by 2050 remains a key target within the Sustainable Development Goals.

On a daily basis, TB causes the death of more than 4100 individuals and infects nearly 28,000 people worldwide, despite being both preventable and curable. Efforts to combat the disease have saved an estimated 66 million lives since 2000. However, the COVID-19 pandemic has disrupted these gains, reversing years of progress, and in 2020, TB-related mortality saw an unprecedented increase for the first time in over a decade [6].

In the Kingdom of Saudi Arabia (KSA), tuberculosis continues to pose significant public health challenges. Due to the country’s role as a hub for international pilgrims and the substantial expatriate population, KSA faces heightened risks of both domestic transmission and the potential emergence of multidrug-resistant TB strains [7]. The WHO underscores the importance of protecting patient confidentiality when collecting risk factor data during TB prevalence studies to ensure ethical standards are maintained [8].

This study aims to identify the key risk factors associated with pulmonary tuberculosis among patients registered in the Aseer Region of Saudi Arabia.

## 2. Materials and Methods

### 2.1. Study Population

A case–control study design was followed in the Aseer Region, Saudi Arabia. All currently registered active TB cases at the Tuberculosis Control Unit, Abha Health Sector, Abha City (n = 130), constituted the study population.

### 2.2. Sample Size

The minimum sample size for included active TB cases was determined to be 98 TB cases using the Raosoft Sample Size Calculator website (http://www.raosoft.com/samplesize.html (accessed on 22 October 2025)), according to the following data:

- Acceptable margin of error: 5%;
- Confidence level: 95%;
- Population size: 130;
- Response distribution: 50%.

However, the study sample was increased by 10% to include 105 active pulmonary TB cases.

### 2.3. Selection of Cases

Currently active pulmonary TB patients registered at the Tuberculosis Control Unit, Aseer Health Cluster, agreed to participate in this study.

### 2.4. Selection of Control Subjects

A total of 143 healthy control subjects were randomly recruited from those attending the vaccination units at several Primary Healthcare Centers (PHCCs) in Aseer Region (i.e., Al-Mansak PHCC, Al-Mowathafeen PHCC, Mahayel PHCC, Sultan City PHCC, Bareq PHCC, and Om Sarar PHCC). These six PHCCs were selected following a simple random sampling technique from a total of 42 PHCCs in the Aseer Region.

### 2.5. Data Collection Tool

Based on a relevant review of the literature, a study questionnaire was designed by the researchers (in Arabic and English), which was used for interviewing participants in the study and control groups. A pretest with 25 randomly selected individuals assessed validity, reliability, applicability, and average completion time, yielding a Cronbach’s alpha of 0.76.

The study questionnaire includes 21 questions (for all participants) about personal characteristics (e.g., age, gender, nationality, education, employment, monthly income, etc.), in addition to the current medical history (e.g., diabetes, renal diseases, and HIV/AIDS) and family history of TB. Moreover, questions were directed to active pulmonary TB cases about their duration of their TB and the follow-up of their condition. Body mass index (BMI) was classified as underweight (<18.5), normal (18.5–24.9), and overweight (≥25).

An income below approximately SAR 5000 per month was commonly considered indicative of low income or poverty, especially when evaluating factors like the high cost of living, housing expenses, and basic needs in the country.

### 2.6. Ethical Considerations

The ethical approvals were obtained from the Institutional Review Board (IRB) at the General Directorate of Health in Aseer Region. Written informed consent was obtained from participants. All participants remained anonymous, and data were treated with full confidentiality.

### 2.7. Time Frame

The study commenced in November 2024, and the data was collected in the period till 1 April 2025.

### 2.8. Data Analysis

Collected data were analyzed using the Statistical Package for Social Sciences (IBM Corp., Armonk, NY, USA), SPSS, version 25). Frequencies and percentages were used to describe qualitative variables. The Chi-square (χ^2^) test was used to compare the sociodemographic characteristics between the study groups. Binary logistic regression was applied to identify risk factors for pulmonary TB. *p*-values less than 0.05 were considered statistically significant.

## 3. Results

Table 1 shows that most participant TB cases were diagnosed during 2024 (95, 9.5%), while 10 cases (9.5%) were diagnosed during 2023. The majority of cases were new (100, 95.2%), while only five cases were relapses (4.8%). A total of 42 TB cases (40%) were living in Abha City, while 45 cases (42.9%) were living in Khamis Mushayt City, and 18 cases (17.1%) were living in other places in Aseer Region.

**Figure 1:**
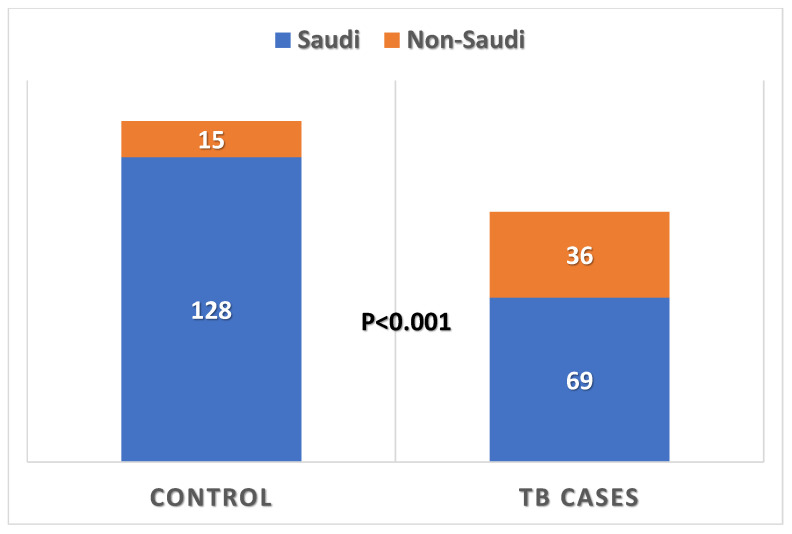
Nationality of pulmonary TB patients compared with those of control subjects.

Table 2 shows that participants included 105 TB patients (study group) and 143 subjects (control group). Both groups differed significantly according to their nationality, with 65.7% being Saudi nationals in the TB group compared with 89.5% (*p* < 0.001, Figure 1).

**Figure 2:**
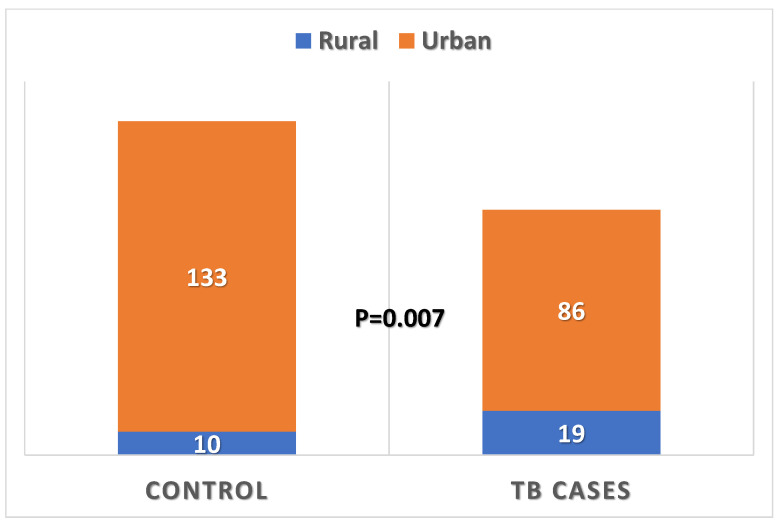
Residence of pulmonary TB patients compared with those of control subjects.

**Figure 3:**
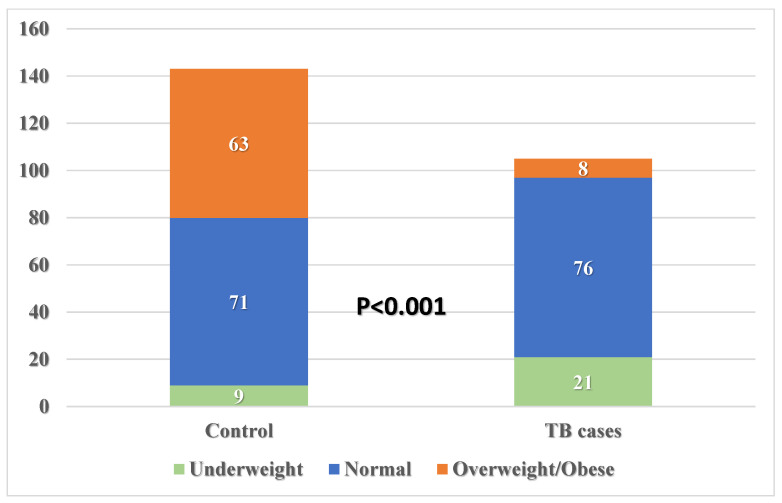
Body mass index grades of pulmonary TB patients compared with those of control subjects.

Participants differed significantly according to their residence, with 18.1% in the TB group living in rural areas compared with only 7% in the control group (*p* = 0.007) (Figure 2). Also, participants differed significantly according to their educational status, with 27.6% being university graduates in the TB group compared with 53.8% in the control group (*p* < 0.001). More than half of the TB patients were single (70.5%), compared with 44.1% in the control group. Both groups differed significantly according to their marital status (with 70.5% of TB patients being single, compared with only 29.5% of control subjects, *p* < 0.001). Moreover, both groups differed significantly according to their employment status and monthly income (*p* < 0.001 for both variables), with more unemployment among TB patients than controls (32.4% and 13.3%, respectively) and more minimal monthly income (SAR < 5000) among TB patients than controls (80% and 44.1%, respectively). One-fifth of the participant TB patients were underweight (20%), compared with only 6.3% in the control group (*p* < 0.001), as shown in Figure 3. Both groups differed significantly according to their marital status (*p* < 0.001). There was a significantly higher positive family history of TB among patients than control subjects (14.3% and 5.6%, respectively, *p* = 0.020). Both groups did not differ significantly according to their age, sex, family size, or smoking status.

Table 3 shows that participants in both groups differed significantly according to their residence (*p* = 0.007), with more rural residence among TB patients than controls (18.1% and 7%, respectively). Also, participants differed significantly according to their type of houses (*p* = 0.001), with 84.8% of TB patients living in apartments, compared to 68.5% of the control group. Significantly more houses were leased by participants in the control group than in the TB group (49.4% and 32.4%, respectively, *p* = 0.009). Significantly more TB patients were living in the basement than the control group (7.6% and 1.4%, respectively, *p* = 0.002). However, both groups did not differ significantly according to the number of bedrooms, number of windows, or number of persons using a bedroom.

Table 4 shows that associated comorbidities among control subjects and TB cases mainly included diabetes (10.5% and 9.5%, respectively), hypertension (9.1% and 3.8%, respectively), respiratory diseases (2.8% and 1%, respectively), and arthritis (2.1% and 1%, respectively). HIV/AIDS affected one TB case. There were no statistically significant differences between participants in the two groups according to associated comorbidities.

Table 5 shows the binary logistic regression model of the possible risk factors related to TB. The odds ratios were highest for participants’ nationality (2.421, 95% CI: 1.018–5.754). However, nationality, residence, and body mass index were significant independent risk factors for pulmonary TB (*p* = 0.045, *p* = 0.032, and *p* < 0.001, respectively).

## 4. Discussion

Tuberculosis is one of the main causes of death from infectious chronic diseases [9]. The Gulf region, including Saudi Arabia, experiences a unique epidemiological profile of TB as a result of several factors, including the high immigration rates and rapid urbanization [10]. Although the incidence of TB in the Gulf countries is relatively low compared to global averages, it is still a considerable public health concern, mainly due to the high flow of workers coming from TB-endemic countries [11].

Therefore, the present study followed a case–control research design to explore the descriptive epidemiology of tuberculosis (according to person, place, and time) among patients in the Aseer Region, Saudi Arabia.

The present study included 105 active pulmonary TB cases. According to their place of residence, 42 cases were living in Abha City, 45 cases were living in Khamis Mushayt City, and 18 cases were living in other places within the Aseer Region. Most cases were diagnosed during the last year (i.e., 2024), while only 10 cases were diagnosed during 2023. Moreover, the majority of TB cases were new, while only five cases were relapses. Multivariate analysis assessed poor adherence to treatment, development of drug resistance, or other treatment-related challenges. In KSA, there has been a reported steady decline in TB incidence. This decline has been attributed to the implementation of vaccination programs and improved access to health services. Despite being described as a “low-to-middle TB burden country”, specific features may challenge the effectiveness of the Saudi Arabia strategy, namely, the continued influx of millions of people coming for Omra and Haj, in addition to the increasing population mobility, which promotes the spread of TB [12].

Xi et al. [13] argued that multidrug-resistant TB significantly contributes to the treatment failure of newly diagnosed TB patients. It is associated with high case fatality rates. Therefore, early detection of drug resistance and the successful control of this condition are crucial steps in the management of multidrug-resistant TB. Chen et al. [14] added that multidrug-resistant TB frequently results in protracted treatment, increased costs, and unfavorable reactions, posing challenges for managing relapses and difficult-to-treat cases.

Pasipanodya et al. [15] stressed that ensuring adherence to standardized treatment regimens is a cornerstone of multidrug-resistant TB prevention. Directly Observed Therapy remains a globally recommended approach to support adherence, particularly in high-burden settings. In order to reduce vulnerability to TB infection, it is important to address social determinants of health, such as malnutrition, crowded living conditions, and HIV co-infection. Integrated care models that combine TB, HIV, and primary healthcare services have demonstrated improved outcomes in high-risk populations [16].

It is to be noted that the Aseer Region is characterized by being a high-altitude province, located at 2000–2500 m above sea level. Alawi et al. [12] reported that the incidence of TB is relatively low in the Aseer Region (5.07 per 100,000 population in 2019), while in the same year, the incidence in the neighboring province, Jazan, was several times higher (16.55 per 100,000 population).

Pal et al. [17] noted that the prevalence rates of TB are usually lower in cities located at high altitudes. Padilla et al. [18] stated that the low PO_2_ at high altitudes inhibits the growth and survival of *M. tuberculosis* bacilli. Also, low PO_2_ seems to lower the virulence of TB bacilli by limiting their ability to multiply and cause active disease. In the present study, univariate analysis identified several significant person-related risk factors for pulmonary TB, including nationality, educational status, employment status, monthly income, marital status, family history of TB, and body mass index. The higher proportion of non-Saudi citizens among TB patients likely reflects migration patterns and living conditions rather than nationality itself as an independent risk factor.

The higher prevalence of pulmonary TB among non-Saudi than Saudi participants may be confounded by the higher socioeconomic level enjoyed by the Saudi nationals. This may also be confirmed by the findings that TB patients were significantly less university-educated, significantly less employed, and had significantly less monthly income.

Most pulmonary TB patients were unmarried, likely due to lower income. They also had a higher prevalence of positive family history, indicating TB’s high infectivity, and one-fifth were underweight, more so than controls. Multivariate analysis identified nationality, residence, and BMI as the key independent factors associated with pulmonary TB. Low BMI may reflect weight loss caused by active TB rather than being a risk factor itself [19]. This exemplifies reverse causality, where the disease leads to reduced weight, highlighting the need to interpret low BMI carefully in epidemiological studies.

Although variables like marital status, education, and income showed significant differences in univariate analysis, they did not remain significant in the multivariate logistic regression. Confounding factors, collinearity among variables, or interaction effects may have influenced these results. These findings are in accordance with those reported by several national and international studies. Alrajhi and Al-Barrak reported that more than half of the TB patients in Saudi Arabia were non-Saudis [20]. Similarly, Alawi et al. [12] reported that the incidence of TB was significantly higher among non-Saudis (*p* < 0.001). Almutairi et al. stressed the importance of checking the family history of TB patients. They reported that once a case of TB is identified, their contacts must be immediately screened, including all family members, who might be the source of TB infection [21].

In the Aseer Region, Alshahrani et al. reported that risk factors for TB included secondary level of education, rural residence, being divorced, having a low monthly income, and a family size of more than six members [22]. Also, Kapilawanse et al. reported that risk factors for TB include unemployment and low income [23]. Tesema et al. stressed that independent risk factors of TB include low educational levels and a positive family history of TB [24]. Duarte et al. listed that socioeconomic risk factors associated with TB include limited education, low income, and unemployment [25].

This study showed that the most frequently associated comorbidity among pulmonary TB cases and the comparison group was diabetes. Moreover, one of the pulmonary TB cases was positive for HIV/AIDS.

Akashanand et al. added that metabolic risk factors, e.g., diabetes mellitus, are important risk factors that significantly contribute to TB mortality. They explained that the interaction between metabolic disorders and TB leads to impaired immune responses. Moreover, hyperglycemia also heightens inflammatory responses by promoting advanced glycation end products [26].

Similar prevalence of comorbidities such as diabetes and hypertension among controls and TB cases, with no significant differences, was observed in our study. This aligns with global and regional data indicating that while these conditions are common among TB patients, their prevalence often does not significantly differ from controls in various settings [26,27].

Alqadasi et al. [27] argued that, in the Gulf Cooperation Council countries, including Saudi Arabia, the rapid urbanization and economic growth have considerably fostered among their population’s sedentary lifestyles and high-calorie, low-nutrition diets. It is to be noted that, although the rich economy combats many socioeconomic challenges, it encourages several comorbidities, such as diabetes, which increases the risk for TB [28]. Other risk factors for TB included comorbidities like HIV, which diminish the immune function and increase TB risk [24]. Findings of the present study showed that pulmonary TB patients were significantly more residents of rural areas than controls. Moreover, the majority of pulmonary TB patients were living in apartments, which were mainly rented. The basement was more commonly inhabited by pulmonary TB patients than the control subjects. The notable association between basement residence and increased TB risk (*p* = 0.002) underscores the need for focused attention on this living environment. Basements often feature poor ventilation, higher humidity levels, and limited natural light, all of which can contribute to conditions conducive to TB transmission. Additionally, urban overcrowding frequently concentrates vulnerable populations in basement units, further elevating risk. Sulidah et al. stated that home environmental conditions are significant risk factors associated with TB transmission, including residential overcrowding and ventilation [28]. Therefore, improving the home physical environment is necessary for controlling TB transmission. Duarte et al. added that risk factors for TB include poor housing with overcrowding and poor ventilation [25].

Tesema et al. noted that independent risk factors of TB include overcrowded households, limited bedroom space, and the absence of windows [24]. Also, Kapilawanse et al. reported that risk factors for TB include overcrowding [23].

Although preventable and treatable, TB was the second leading cause of death from a single infectious agent in 2022, after COVID-19 and ahead of HIV/AIDS. Ending the TB epidemic requires urgent, coordinated action in line with commitments made at the September 2023 UN meeting [29]. The Essentials emphasize that this involves comprehensive national strategies: expanding healthcare services (Pillar 1), promoting cross-sector collaboration for TB-sensitive programs (Pillar 2), and investing in research to develop innovative tools (Pillar 3) [30].

This study provides valuable initial insights and demonstrates the feasibility of data collection. It also aids in hypothesis generation and reflects a rigorous, transparent approach by acknowledging the following limitations. We recognize that the relatively small sample size of 105 TB cases and 143 controls limits the statistical power and robustness of our conclusions. As a pilot study, our primary aim was to explore potential associations and generate hypotheses to inform future, larger-scale research.

The selection of controls from vaccination units was driven by logistical considerations but may limit their representativeness of the general population, potentially introducing control group selection bias. Future studies should utilize more representative control groups to enhance comparability. However, this approach has advantages: vaccination clinics are easy to access, making recruitment faster. Also, controls were selected during the same time period as the cases, which helps reduce temporal bias. Additionally, important determinants such as HIV status, household crowding, substance use, and corticosteroid therapy were not included in our regression model. The loss of significance for certain variables in multivariable analysis may be due to confounding effects and collinearity. Potential biases—including selection bias, recall bias, and residual confounding—may also influence our findings due to the omission of relevant variables.

We acknowledge that variables like low education, marital status, and income may serve as proxies for broader social disadvantages rather than direct causes. Future research should explore these relationships further, using longitudinal or experimental designs to better understand underlying mechanisms and inform targeted interventions. Including these factors in future research, along with more in-depth analyses of comorbidities like diabetes and HIV (e.g., adjusted or stratified analyses), would strengthen the validity and interpretability of the results. We acknowledge that larger studies, such as those by WHO, provide more comprehensive data on social determinants of tuberculosis. Overall, our findings should be considered preliminary, underscoring the need for larger, more representative studies to confirm these associations. Nonetheless, the study offers valuable initial insights into potential social determinants of pulmonary TB in the region, highlighting important areas for future research.

## 5. Conclusions

To strengthen public health strategies in the Aseer Region, targeted interventions such as migrant screening programs, nutritional support initiatives, and housing improvements should be prioritized. The current study highlights that active pulmonary TB cases often involve non-Saudi individuals, those with lower educational levels, unmarried and unemployed persons, with low income, positive family history of TB, and underweight status. Diabetes mellitus emerges as the most common comorbidity, and HIV/AIDS remains a notable co-infection.

Risk factors linked to environmental conditions include residing in rural areas and living in basement apartments of rented houses. While most pulmonary TB cases resolve within one year, some may experience relapses; hence, adherence to standardized treatment regimens is essential to prevent the development of multidrug-resistant TB.

Reducing vulnerability to TB infection necessitates addressing social determinants such as malnutrition, crowded living environments, and HIV co-infection. Pre-placement screening—particularly for non-Saudi workers from endemic regions—should be reinforced. Public awareness campaigns targeting high-risk rural populations are vital for prevention. Moreover, implementing integrated care models that combine TB, HIV, and primary healthcare services has been shown to improve outcomes among high-risk groups.

## Figures and Tables

**Table 1:** Characteristics of participant pulmonary TB patients (n = 105).

Characteristics	Frequency	Percent
Year of diagnosis		
2024	95	90.5
2023	10	9.5
Type of TB patient		
New	100	95.2
Relapse	5	4.8
Place of residence		
Abha City	42	40.0
Khamis Mushayt City	45	42.9
Others	18	17.1

**Table 2:** Characteristics of participants.

		Control (n = 143)		TB Cases (n = 105)		*p*
Characteristics		No.	%	No.	%	Value
Age groups	<20	9	6.3	6	5.7	
(in years)	20–29	48	33.6	45	42.9	
	30–39	44	30.8	28	26.7	0.658
	40–49	28	19.6	16	15.2	
	50+	14	9.8	10	9.5	
Sex	Male	100	69.9	83	79.0	
	Female	43	30.1	22	21.0	0.107
Nationality	Saudi	128	89.5	69	65.7	
	Non-Saudi	15	10.5	36	34.3	<0.001 †
Residence	Rural	10	7.0	19	18.1	
	Urban	133	93.0	86	81.9	0.007 †
Marital status	Single	63	44.1	74	70.5	
	Married	80	55.9	31	29.5	<0.001 †
Family size	<3	25	17.5	10	9.5	
	3–4	22	15.4	28	26.7	
	5–6	65	45.5	44	41.9	0.082
	7+	31	21.7	23	21.9	
Family History	Yes	8	5.6	15	14.3	
of TB	No	135	94.4	90	85.7	0.020 †
Educational	Illiterate	5	3.5	6	5.8	
Status	Primary	1	0.7	6	5.7	
	Intermediate	9	6.3	9	8.6	<0.001 †
	Secondary	51	35.7	55	52.4	
	University	77	53.8	29	27.6	
Employment	Unemployed	19	13.3	34	32.4	
Status	Student	28	19.6	9	8.6	
	Governmental job	67	46.9	14	13.3	<0.001 †
	Private job	24	16.8	45	42.9	
	Retired	5	3.5	3	2.9	
Monthly	SAR < 5000	63	44.1	84	80.0	
Income	SAR 5000–10,000	36	25.2	12	11.4	<0.001 †
	SAR > 10,000	44	30.8	9	8.6	
Smoking Status	Smoker	27	18.9	22	21.0	
	Non-Smoker	96	67.1	72	68.6	0.688
	Ex-Smoker	20	14.0	11	10.5	
Body Mass	Underweight	9	6.3	21	20.0	
Index (BMI)	Normal weight	71	49.7	76	72.4	<0.001 †
Grades	Overweight/obese	63	44.1	8	7.6	

† Statistically significant.

**Table 3:** Participants’ home environment conditions.

		Control (n = 143)		TB Cases (n = 105)		*p*
Residential and Housing Conditions		No.	%	No.	%	Value
Residence	Rural	10	7.0	19	18.1	
	Urban	133	93.0	86	81.9	0.007 †
Housing type	Apartment	98	68.5	89	84.8	
	Building	21	14.7	13	12.4	0.001 †
	Villa	24	16.8	3	2.9	
Housing	Lease	70	49.0	34	32.4	
condition	Rented	73	51.0	71	67.6	0.009 †
Floor of	Ground	46	32.2	49	46.7	
residence	First	46	32.2	28	26,7	
	Second or higher	48	34.3	29	19.0	0.002 †
	Basement	2	1.4	8	7.6	
No. of	1	15	10.5	15	14.3	
bedrooms	2	33	23.1	25	23.8	0.629
	3+	95	66.4	65	61.9	
No. of	0	0	0.0	2	1.9	
windows	1	88	61.5	71	67.6	0.124
	2	55	38.5	32	30.5	
No. of persons	One	44	30.8	42	40.0	
using a bedroom	More than one	99	69.2	63	60.0	0.131

† Statistically significant.

**Table 4:** Participants’ associated comorbidities.

	Control (n = 143)		TB Cases (n = 105)		*p*
Comorbidities	No.	%	No.	%	Value
Diabetes	15	10.5	10	9.5	0.803
Hypertension	13	9.1	4	3.8	0.104
Respiratory disease	4	2.8	1	1.0	0.295
Arthritis	3	2.1	1	1.0	0.434
Chronic renal disease	1	0.7	1	1.0	0.669
Coronary heart disease	1	0.7	0	0.0	0.391
HIV/AIDS	0	0.0	1	1.0	0.423

**Table 5:** Binary logistic regression for possible risk factors for pulmonary TB.

Independent				Exp	*p*	95% CI for Exp (B)	
Variables	B	S.E.	Wald	(B)	Value	Lower	Upper
Nationality	0.884	0.442	4.005	2.421	0.045 †	1.018	5.754
Education	−0.124	0.179	0.478	0.884	0.489	0.623	1.255
Marital	−0.307	0.373	0.677	0.736	0.411	0.354	1.529
Income	−0.483	0.277	3.026	0.617	0.082	0.358	1.063
Smoking status	−0.376	0.282	1.774	0.687	0.183	0.395	1.194
Residence	−1.186	0.553	4.598	0.305	0.032 †	0.103	0.903
Floor	0.033	0.095	0.118	1.033	0.731	0.858	1.244
Family size	0.030	0.189	0.026	1.031	0.872	0.712	1.494
Bedrooms	0.079	0.268	0.086	1.082	0.769	0.640	1.829
Body mass index	−1.203	0.306	15.450	0.300	<0.001 †	0.165	0.547
Job	0.140	0.096	2.131	1.150	0.144	0.953	1.387
Constant	3.996	1.687	5.612	54.355	0.018		

† Statistically significant.

## Data Availability

The data presented in this study are available on request from the corresponding author due to privacy restrictions.

## References

[1] Barberis I., Bragazzi N.L., Galluzzo L., Martini M. (2017). The history of tuberculosis: From the first historical records to the isolation of Koch’s bacillus. J. Prev. Med. Hyg.

[2] Duko B., Bedaso A., Ayano G., Yohannis Z. (2019). Perceived Stigma and Associated Factors among Patient with Tuberculosis, Wolaita Sodo, Ethiopia: Cross-Sectional Study. Tuberc. Res. Treat..

[3] Saati A.A., Khurram M., Faidah H., Haseeb A., Iriti M. (2021). A Saudi Arabian Public Health Perspective of Tuberculosis. Int. J. Environ. Res. Public Health.

[4] Jurado L.F., Pinzón B., De La Rosa-Noriega Z.R., Matijasevic E., del Pilar López-Panqueva R. (2019). Peritoneal tuberculosis in a health-care worker, radio-pathological assessment and diagnosis, a case report. Radiol. Infect. Dis..

[5] Ashaba C., Musoke D., Wafula S.T., Konde-Lule J. (2021). Stigma among tuberculosis patients and associated factors in urban slum populations in Uganda. Afr. Health Sci..

[6] PAHO World Tuberculosis Day 2022. Invest to End TB. Save Lives. The Pan American Health Organization (PAHO)..

[7] Ahmed M.M., Velayati A.A., Mohammed S.H. (2016). Epidemiology of multidrug-resistant, extensively drug resistant, and totally drug resistant tuberculosis in Middle East countries. Int. J. Mycobacteriol..

[8] WHO (2011). Tuberculosis Prevalence Surveys: A Handbook.

[9] Udoakang A.J., Djomkam Zune A.L., Tapela K., Nganyewo N.N., Olisaka F.N., Anyigba C.A., Tawiah-Eshun S., Owusu I.A., Paemka L., Awandare G.A. (2023). The COVID-19, tuberculosis and HIV/AIDS: Ménage à Trois. Front. Immunol..

[10] Jackson S., Kabir Z., Comiskey C. (2021). Effects of migration on tuberculosis epidemiological indicators in low and medium tuberculosis incidence countries: A systematic review. J. Clin. Tuberc. Other Mycobact. Dis..

[11] Abdelwahab S.I., Taha M.M.E., Albasheer O., Alharbi A., Ahmed A.A., Abdelmola A., Ali S.A., El Hassan L.A., Darraj M., Mohamed A.H. (2024). Tuberculosis research advances and future trends: A bibliometric knowledge mapping approach. Medicine.

[12] Alawi M.M., Alserehi H.A., Ali A.O., Albalawi A.M., Alanizi M.K., Nabet F.M., Alkamaly M.A., Assiri A.M., Jokhdar H., Qutub M.O. (2024). Epidemiology of tuberculosis in Saudi Arabia following the implementation of end tuberculosis strategy: Analysis of the surveillance data 2015–2019. Saudi Med. J..

[13] Xi Y., Zhang W., Qiao R.-J., Tang J. (2022). Risk factors for multidrug-resistant tuberculosis: A worldwide systematic review and meta-analysis. PLoS ONE.

[14] Chen H., Tong Z., Zhong J., Zeng Q., Shen B., Qian F., Xiao X. (2025). Epidemiological characteristics and risk factors analysis of multidrug-resistant tuberculosis among tuberculosis population in Huzhou City, Eastern China. Open Life Sci..

[15] Pasipanodya J.G., Srivastava S., Gumbo T. (2012). Meta-analysis of clinical studies supports the pharmacokinetic variability hypothesis for acquired drug resistance and failure of antituberculosis therapy. Clin. Infect. Dis..

[16] Tiberi S., du Plessis N., Walzl G., Vjecha M.J., Rao M., Ntoumi F., Mfinanga S., Kapata N., Mwaba P., McHugh T.D. (2018). Tuberculosis: Progress and advances in development of new drugs, treatment regimens, and host-directed therapies. Lancet Infect. Dis..

[17] Pal A., Acharya T., Hamal K., Acharya N., Shah K.B. (2023). Prevalence of Tuberculosis Among High Altitude Residents of Nepal. Nepal Med. Jor..

[18] Pérez-Padilla R., Franco-Marina F. (2004). The impact of altitude on mortality from tuberculosis and pneumonia. Int. J. Tuberc. Lung Dis..

[19] Narasimhan P., Wood J., MacIntyre C.R., Mathai D. (2013). Risk factors for tuberculosis. Pulm. Med..

[20] Alrajhi A.A., Al-Barrak A.M., Madkour T.M.M. (2004). Epidemiology of Tuberculosis in Saudi Arabia.

[21] Almutairi F.M., Tayeb T., Alhakeem R., bin Saeed A., Assiri A., McNabb S.J. (2018). Distribution and determinants of tuberculosis in the Kingdom of Saudi Arabia from 2005 to 2012. J. Epidemiol. Glob. Health.

[22] Alshahrani F., Zafar M., Omer E.O. (2021). Lifestyle risk factors associated with tuberculosis patients in Asir region of Saudi Arabia. Int. J. Prev. Med..

[23] Kapilawanse S., Bichha R.P., Samaraweera S., Pallewatte N., Vitharana H., Jayakody W., Gangathesewaran M. (2018). Tuberculosis Among Young People on Rise in Sri-Lanka (An Analysis of Trend and Associated Factors). SAARC J. Tuberc. Lung Dis. HIV/AIDS.

[24] Tesema C., Tadesse T., Gebrehiwot M., Tsegaw A., Weldegebreal F. (2015). Environmental and host-related determinants of tuberculosis in Metema district, north-West Ethiopia. Drug Health Patient Saf..

[25] Duarte R., Lönnroth K., Carvalho C., Lima F., Carvalho A., Muñoz-Torrico M., Centis R. (2018). Tuberculosis, social determinants and co-morbidities (including HIV). Pulmonology.

[26] Akashanand, Samal S.K., Gaidhane S., Jena D., Roopashree R., Kaur M., Nathiya D., Sharma A., Prasad G.S., Sinha A. (2025). Epidemiological trends and forecasting of tuberculosis burden in the Gulf Cooperation Council countries: Evidence from global burden of disease 1990–2021. J. Infect. Public Health.

[27] Alqadasi E.T., Chamroonsawasdi K., Saejeng K., Nagi M.A. (2024). Burden of non-communicable diseases in Health Council of Gulf Cooperation (GCC) countries. J. Taibah Univ. Med. Sci..

[28] Sulidah S., Irwan M., Elmania E., Fadlilah S., Hamdani-Rahil N., Nugroho A. (2024). Home environment as a risk factor for increased incidence of tuberculosis: A case-control study. Cienc. Enferm..

[29] World Health Organization (2022). Implementing the End TB Strategy: The Essentials, 2022 Update.

[30] World Health Organization (2023). Global Tuberculosis Report 2023.

